# Deciphering Müller cell heterogeneity signatures in diabetic retinopathy across species: an integrative single-cell analysis

**DOI:** 10.1186/s40001-024-01847-y

**Published:** 2024-05-03

**Authors:** Xiyuan Deng, Ya Mo, Xiuying Zhu

**Affiliations:** 1 Chengdu University of Traditional Chinese Medicine, Chengdu, China; 2 Hospital of Chengdu University of Traditional Chinese Medicine, Chengdu, China

**Keywords:** Diabetic retinopathy, Single-cell transcriptomics, Müller cell, RHO

## Abstract

Diabetic retinopathy (DR), a leading cause of visual impairment, demands a profound comprehension of its cellular mechanisms to formulate effective therapeutic strategies. Our study presentes a comprehensive single-cell analysis elucidating the intricate landscape of Müller cells within DR, emphasizing their nuanced involvement. Utilizing scRNA-seq data from both Sprague–Dawley rat models and human patients, we delineated distinct Müller cell clusters and their corresponding gene expression profiles. These findings were further validated through differential gene expression analysis utilizing human transcriptomic data. Notably, certain Müller cell clusters displayed upregulation of the Rho gene, implying a phagocytic response to damaged photoreceptors within the DR microenvironment. This phenomenon was consistently observed across species. Additionally, the co-expression patterns of RHO and PDE6G within Müller cell clusters provided compelling evidence supporting their potential role in maintaining retinal integrity during DR. Our results offer novel insights into the cellular dynamics of DR and underscore Müller cells as promising therapeutic targets for preserving vision in retinal disorders induced by diabetes.

**Supplementary Information:**

The online version contains supplementary material available at 10.1186/s40001-024-01847-y.

## Introduction

Diabetic retinopathy (DR) is a severe complication of diabetes mellitus, posing a significant threat to vision and often leading to visual impairment and blindness, particularly affecting individuals of working age [1]. This condition is characterized by progressive damage to retinal microvasculature, neurodegeneration, and inflammation, ultimately resulting in vision loss if not adequately managed [2].

Müller cells, serving as the primary glial cells in the retina, play crucial roles in maintaining retinal homeostasis and supporting neuronal and vascular functions [3, 4]. They offer structural reinforcement, regulate extracellular ions and neurotransmitters, and actively participate in metabolic and antioxidant processes. Nevertheless, prolonged exposure to high blood sugar levels can lead to alterations in both the functional and molecular aspects of Müller cells [5–7].

To comprehend the molecular mechanisms underlying Müller cell dysfunction in DR, it is crucial to utilize advanced techniques capable of capturing both the cellular heterogeneity and dynamic changes within specific cell populations. While conventional methods like bulk transcriptomics provide valuable insights into overall gene expression alterations linked to DR, they often lack the ability to address the complexities of cellular diversity and may obscure critical cellular pathways.

Recent advancements in single-cell transcriptomics have brought a revolutionary impact on our ability to study complex biological systems with unprecedented resolution. Through the profiling of individual cell transcriptomes, this technology enables the identification of specific cell subtypes, analysis of cellular diversity, and elucidation of cellular dynamics during disease progression [8]. Utilizing single-cell transcriptomics to investigate Müller cells in the context of DR offers immense potential in discovering new molecular markers and gaining insights into the intercellular communication networks that underlie the development of the disease.

In this study, our objective was to elucidate the pivotal roles played by Müller cells within the diabetic retinopathy (DR) microenvironment. We aimed to unravel the molecular complexities of Müller cell heterogeneity and their dynamic communication patterns throughout the progression of DR. Leveraging cross-species single-cell transcriptomic analysis and validating interactions through established databases, we sought to decipher the intricate cellular communications mediated by Müller cells. Our focus was particularly on exploring co-expression patterns of relevant genes to enhance our understanding of Müller cells' potential phagocytic and supportive functions in DR. This investigation is expected to provide new molecular insights into DR pathogenesis and reveal novel targets for therapeutic intervention that may alter the course of the disease and offer hope for preserving vision in affected individuals.

## Methods

### Data source

The data used in this study were retrieved from three GEO datasets (https://www.ncbi.nlm.nih.gov/geo/) available in the GEO database: GSE209872, GSE137537 and GSE160306. The GSE209872 dataset comprises single-cell sequencing (scRNA-seq) data obtained from retinal specimens of Sprague–Dawley (SD) rats. This dataset includes a total of five samples, including two samples from the control group at 0 weeks and one sample each from the experimental group at 2 weeks, 4 weeks, and 8 weeks after the induction of DR. As for GSE137537, it encompasses a substantial dataset of parallel single-cell RNA sequencing data obtained from the human retina employing two independent platforms. This dataset includes samples from 3 cases of Macula retina (MR) and 3 cases of Peripheral retina (PR). The GSE160306 dataset comprises high-throughput transcriptome sequencing data, annotated with the Illumina HiSeq 4000 platform (GPL20301), from post-mortem human eyes. This dataset encompasses samples from a total of 10 donors, representing different groups, including healthy controls, diabetic individuals, non-proliferative diabetic retinopathy (NPDR), and NPDR/PDR + DME (diabetic macular edema). The tissue samples were collected from both the macula region and the retinal periphery.

### Single-cell sequencing data analysis

To perform data quality control and preprocessing of the scRNA-seq data, we utilized the Seurat package (version 4.3.0) [9]. Initially, we loaded and processed the raw sequencing data and conducted an assessment of basic data quality metrics. Integration of the raw sequencing data from SD rats was performed using the Harmony R package (version 0.1.1) [10]. Subsequently, cells exceeding a mitochondrial gene percentage of 10% or having fewer than 300 detected genes were filtered out to eliminate low-quality cells. Furthermore, a scale factor of 10,000 was applied to normalize all the remaining cells, ensuring comparability across samples. To identify genes differentially expressed between different cell clusters, we used the FindAllMarkers and FindMarkers functions in the Seurat package and set a logFC threshold of 0.25. For the pseudo-time analysis of Müller cells, we employed the Monocle2 package (version 2.24.0) [11], a widely used computational tool specifically designed for inferring and visualizing the developmental trajectories of individual cells based on their gene expression profiles. To investigate the cell–cell interactions between Müller cells and other cell clusters, we utilized the CellChat (version 1.6.1) package [12, 13]. Through the calculation of interaction potential scores, we assessed the strength of cell–cell interactions. This analysis allowed us to identify the most relevant cell types involved in interactions with Müller cells and explore the underlying interaction networks.

### Differential gene expression analysis

We utilized the limma package (version 3.54.0) [14], a widely employed statistical tool for differential expression analysis in transcriptomics research. To determine significant differential expression, we applied stringent statistical thresholds. Genes with a p-value below 0.05 were considered statistically significant, ensuring the reliability of our findings. Additionally, we applied a fold change threshold, requiring a magnitude greater than 1 (|log2 Fold Change (FC)|> 1) to ensure biological relevance. Furthermore, we constructed a gene co-expression regulatory network to investigate the regulatory relationships among genes in DR. This network was constructed by computing Pearson correlations, which gauge both the strength and direction of the linear relationships between pairs of variables.

### Gene name conversion

In this study, we performed gene name conversion between humans and SD rats using the biomaRt package (version 3.54.0) [15]. The biomaRt package provides a comprehensive set of functions for accessing and extracting biological data from online databases, including gene annotation and mapping information. This method highlights the established and widely adopted practice of cross-species gene name conversion in the realm of bioinformatics research [16].

### Enrichment analysis of gene functions

Gene enrichment analysis was carried out using the DAVID database (https://david.ncifcrf.gov/), specifically utilizing the “Functional Annotation Chart” tool. To mitigate concerns related to multiple testing, we employed Benjamini–Hochberg corrections to control the false discovery rate (FDR). Gene Ontology (GO) and Kyoto Encyclopedia of Genes and Genomes (KEGG) analyses represent established bioinformatics approaches employed to unveil the functional attributes and enrichment of genes implicated in distinct biological processes (BP) and metabolic pathways. GO serves as a standardized annotation system that describing gene functions, processes, and cellular components (CC). Through GO analysis, we can discern significant characteristics of these genes in terms of their involvement in BP, CC, and molecular functions (MF) by comparing the enrichment of various gene sets in the study. In contrast, KEGG is a comprehensive database compiling information about metabolic pathways, signaling pathways, and genes associated with diseases. KEGG analysis allows us to unveil the enrichment of genes within specific signaling pathways, shedding light on their potential roles and mechanisms in relevant BP and disease development.

### Protein–protein interaction analysis

For the identification and visualization of protein–protein interactions, we utilized the STRING database (https://string-db.org/). Specifically, gene names were uploaded to the STRING database to facilitate this analysis. To ensure the high relevance of the interactions displayed, a confidence score threshold of 0.4 was set.

### Statistical analysis

All statistical analyses were performed with R software (version 4.0.3). The correlation between two continuous variables was assessed using Pearson correlation. For all statistical tests conducted within this study, a two-tailed *p*-value below 0.05 was considered indicative of statistical significance for all conducted statistical tests.

## Results

### Single-cell atlas of DR

**Fig. 1:**
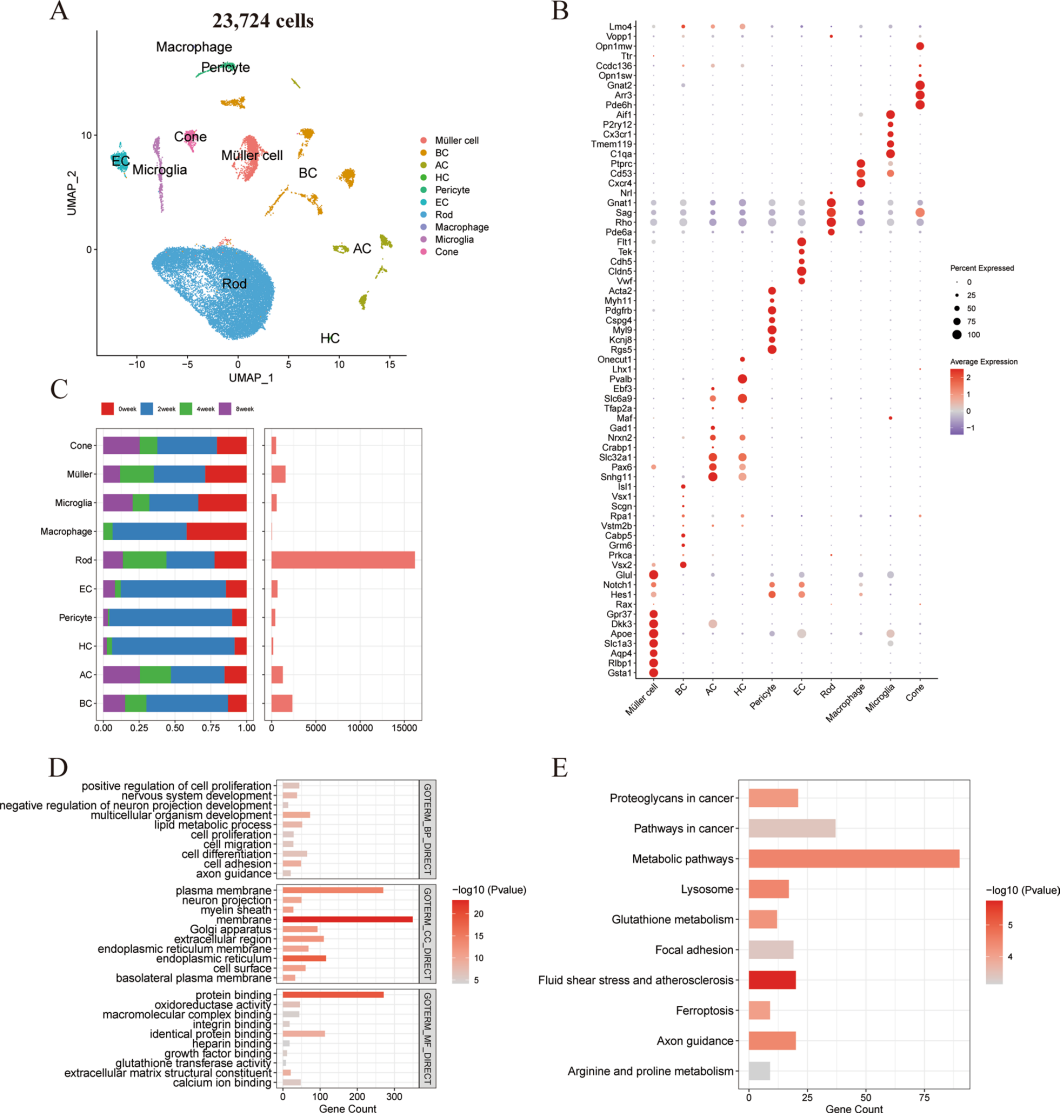
**A** The UMAP plot of scRNA-seq data, colored for the 10 cell clusters. **B** The dotplot heatmap of the marker genes across individual cell clusters. **C** Stacked bar chart representing the proportional composition of various retinal cell types at different time points (0, 2, 4, and 8 weeks). Box plot showing the total counts of each retinal cell type pooled from all time points. **D** Top 10 GO terms in BP, CC and MF. **E** Top 10 KEGG pathways

After performing quality control and preprocessing of the scRNA-seq data, a total of 23,724 cells were obtained for subsequent analysis. To visualize the cell types, we applied Uniform Manifold Approximation and Projection (UMAP) plot analysis to reduce the high-dimensional data. The UMAP plot revealed distinct separation of different cell types, indicating their unique transcriptional profiles (Fig. 1A). Additionally, each cell type was characterized by examining specific marker genes for individual cell clusters. These marker genes exhibited high expression levels in their respective cell types, confirming their identity and providing evidence for the accuracy of the cell type classification (Fig. 1B). By analyzing gene expression patterns, we categorized the cells into 10 distinct types, including rod cells, cone cells, amacrine cells (AC), bipolar cells (BC), Müller cells, microglia, endothelial cells (EC), horizontal cells (HC), macrophages, and pericytes. We then assessed the distribution and prevalence of these cell subtypes. The findings indicated that the rod cell subtype had the highest abundance, followed by the BC, Müller cell, and AC cell subtypes (Fig. 1C).

To gain insights into the functional characteristics of Müller cell subtypes, we conducted a gene differential expression analysis with Seurat. This analysis aimed to investigate the differential expression of genes between Müller cells and other cell subtypes. Subsequently, we performed a gene enrichment analysis on these identified genes, detailed in Additional file 3: Table S1. In terms of BP, there is a pronounced enrichment of genes involved in the positive regulation of cell proliferation, nervous system development, and the negative regulation of neuron projection development. Additionally, processes like lipid metabolic process, cell migration, and differentiation are also notably represented. This suggests a multifaceted role of Müller cells in maintaining retinal structure and function, potentially implicating these cells in both supportive and regulatory capacities in the neural environment. The CC analysis highlights a concentration of genes associated with various structures integral to cell communication and nutrient transport, such as the plasma membrane, neuron projection, myelin sheath, and extracellular region. The presence of genes related to the Golgi apparatus and endoplasmic reticulum membrane underscores the Müller cells' involvement in protein processing and trafficking. MF results exhibit a significant overrepresentation of genes coding for proteins with binding abilities, including protein binding, oxidoreductase activity, and ion binding activities like calcium ion binding, which are critical for cell signaling and metabolic processes. The abundance of genes associated with the extracellular matrix and growth factor binding further emphasizes the role of Müller cells in extracellular matrix remodeling and interaction with growth factors, which are crucial for retinal development and repair mechanisms (Fig. 1D). Additionally, our KEGG pathway analysis identified several significantly enriched pathways, such as metabolic pathways, pathways in cancer, and fluid shear stress and atherosclerosis (Fig. 1E).

### Intercellular signaling and communication network of Müller cells

**Fig. 2:**
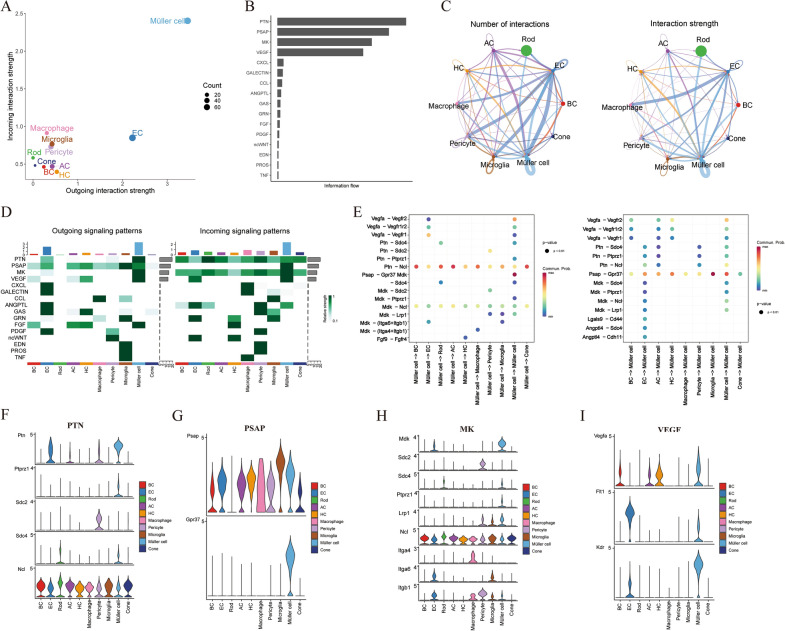
**A** Scatter plot depicting the incoming and outgoing interaction strengths among various cell types. **B** The information flow of various signaling molecules within the retinal microenvironment in the context of diabetic retinopathy. **C** Network analysis of the number/strength of interactions. **D** The heatmap of signaling pathways related to outgoing signaling patterns and incoming signaling patterns. **E** The dot plot of incoming/outcoming communication patterns of Müller cells. **F** The violin plot displaying the expression profiles of key signaling molecules PTN, PSAP, MK, and VEGF across different cell types

To investigate the communication mechanisms of Müller cells with other cell subtypes during the progression of DR, we employed CellChat to construct the communication network between Müller cells and others, as illustrated in Additional file 1: Fig. S1. Our analysis revealed that Müller cells displayed the highest relative signal strength in both incoming and outgoing signaling patterns as depicted in Fig. 2A. This suggests that Müller cells are central to the intercellular communication networks in the DR. Specifically, we observed that pleiotrophin (PTN), prosaposin (PSAP), and midkine (MK) were the most critical signaling pathways in the information flow initiated or received by Müller cells, signifying their essential roles in the progression of DR (Fig. 2B). In the network analysis of the cellular interaction, we observed a substantial degree of complexity and specificity in cell–cell communication. The left panel (Fig. 2C), detailing the number of interactions, shows that Müller cells, microglia, and EC are hubs of cellular interaction within the retinal microenvironment, engaging in numerous contacts with other cell types such as AC, BC, and EC. The right panel, focusing on interaction strength, further elaborated on the quality of these interactions. Here, Müller cells displayed prominent signaling connections, indicating not just frequent but also strong communicative links, which may be pivotal in maintaining retinal homeostasis and responding to pathophysiological conditions. We further presented a detailed analysis of the signaling patterns and ligand–receptor interactions within the retinal microenvironment. Figure 2D elucidates a diverse suite of signaling molecules, encompassing growth factors and inflammatory mediators. Notably, Müller cells were highlighted for their extensive expression of signaling molecules, confirming their central role as previously indicated in the network diagrams. Within these cells, we identified PTN, MK, PSAP, and VEGF as the top signaling pathways. Our focus was narrowed to investigate the pathways predominantly mediated by Müller cells within the communication network. Ptn interacting with its receptor Ncl and Mdk with receptors such as Ncl and Lrp1, were prominent in Müller cell signaling output. Finally, we collectively depict the expression patterns of key signaling molecules (PTN, PSAP, MK, and VEGF) and their receptors across various cell types in the context of DR. Across all cell types, Müller cells consistently showed high expression levels of these signaling molecules, indicating their significant role in intercellular communication within the progression of DR (Fig. 2f–I).

### Temporal dynamics and cellular heterogeneity of Müller cells

**Fig. 3:**
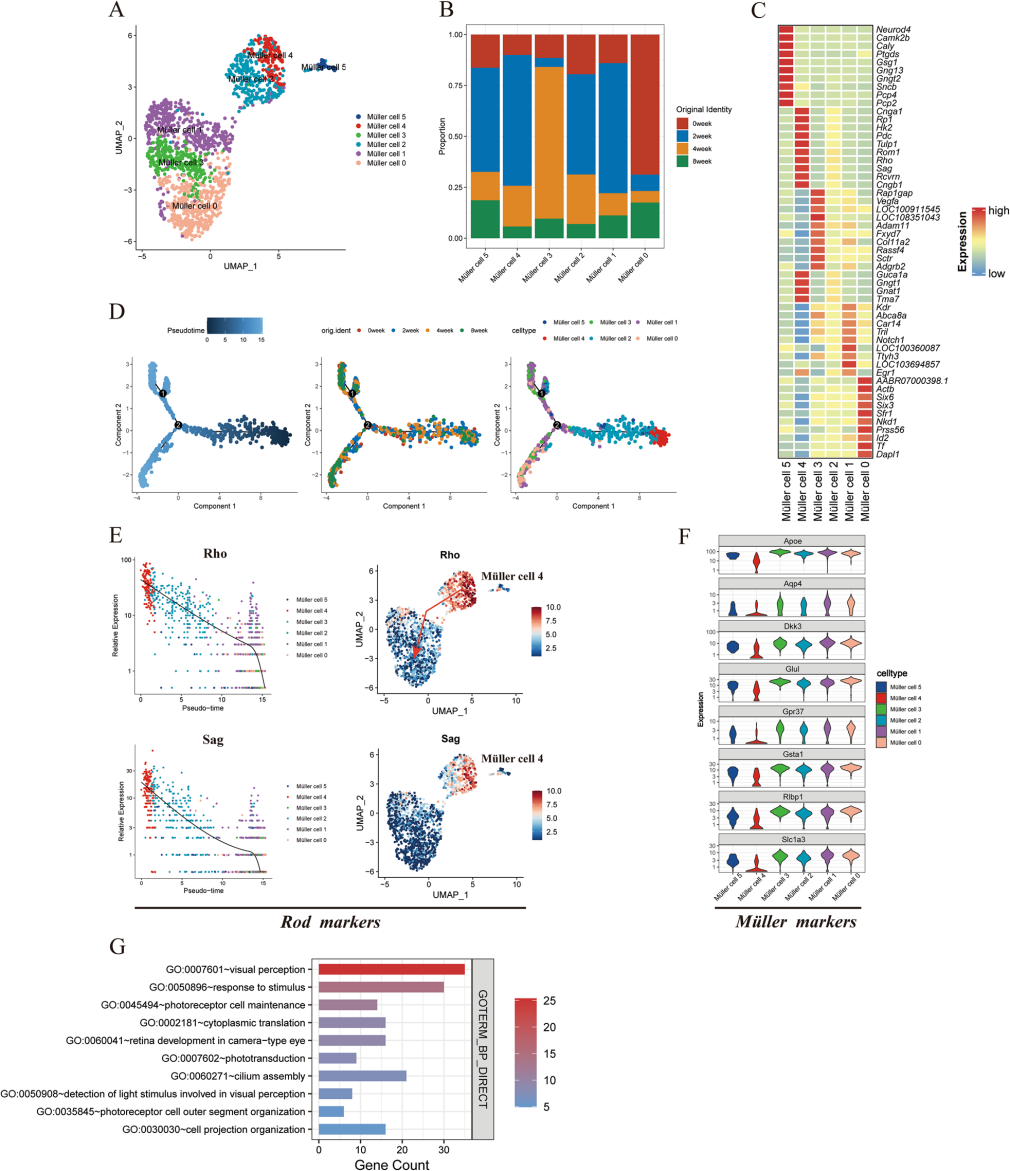
**A** The UMAP plot of Müller cell clusters. **B** Stacked bar plot depicting the proportion of each Müller cell clusters’ original identity at different time points (0 week, 2 weeks, 4 weeks, and 8 weeks). **C** Heatmap of characteristic gene expression across Müller cell clusters. **D** Monocle analysis plot of Müller cells in 2D-PCA space, with a color code for pseudo-time, orig.ident and cell type. **E** Scatter plots illustrating the expression of Rho and Sag, across a pseudo-time trajectory in different Müller cell clusters. **F** Violin plots of Müller cell marker gene expression. **G** Gene Ontology Enrichment Analysis of the characteristic gene of Müller cell 4

Upon analyzing single-cell RNA sequencing data from a rat model, we categorized Müller cells into six distinct subgroups to delineate their dynamic phenotypic changes during the progression of diabetic retinopathy at defined intervals of 0, 2, 4, and 8 weeks, encompassing a total of 1823 Müller cells identified in the dataset (Fig. 3A, B). The clustering of gene expression patterns across the different Müller cell clusters might indicate heterogeneity in the functional states of these cells, possibly reflecting various stages within the DR environment (Fig. 3C). Pseudo-time analysis was then performed on Müller cells (Fig. 3D). We demonstrated the trajectory is bifurcated, indicating two potential differentiation paths that these cells may assume in the DR. When cross-referenced with the temporal data (0 to 8 weeks), the result showed that the Müller cells from the early weeks (0 and 2) were predominantly positioned at the beginning of the trajectory. In contrast, cells from later weeks (4 and 8) progressively occupied the terminal end of the trajectory. This pattern indicated that Müller cells undergo transcriptional changes over time in the DR condition, possibly reflecting the progression of the disease state. The distribution of Müller cell clusters along the trajectory reveals that Müller cell 1 and 0 were predominantly found at the later stages of pseudo-time. Meanwhile, Müller cell 2 and 4 appeared to be more prevalent at the early stages of the trajectory (Fig. 3D). It becomes evident that there is considerable heterogeneity in the transcriptional profiles of Müller cell subtypes during the progression of DR. The bifurcated trajectory and the chronological distribution of cells suggested that Müller cells do not represent a monolithic population but rather undergo diverse transcriptional changes over time, possibly in response to the DR milieu.

We observed an unexpected high expression of the rhodopsin (Rho) within the Müller cell 4. Rhodopsin is typically expressed exclusively in rod photoreceptors, where it serves as a photopigment essential for the capture of light signals. Its expression in Müller cell 4 was indeed surprising, as these glial cells did not normally participate directly in phototransduction. Figure 3E depicts the relative expression of Rho across pseudo-time, showing a significant expression in Müller cell 4, corroborated by the UMAP plot which localizes this expression specifically to this subpopulation. Concurrently, the analysis of rod and Müller cell markers indicated that while Müller cell 4 expresses rod-specific markers like Rho and Sag, it also maintained the expression of typical Müller cell markers such as Glul and Apoe, among others (Fig. 3E–F).

This co-expression of rod and Müller cell markers suggest a phenomenon of cellular interaction or fusion, possibly through phagocytosis of damaged rod cells by Müller cell 4. Phagocytosis is a known response by Müller cells to maintain retinal integrity under stress conditions, such as diabetic retinopathy. Given the context, it is plausible to speculated that Müller cell 4 was actively engaged in the clearance of damaged rods, thus acquiring their markers. The high expression of Rho in Müller cell 4 suggested an adaptation or response mechanism that may be unique to the DR environment. The retention of rod-specific markers within the Müller cell 4 might reflect a protective mechanism aimed at preserving retinal function in the face of diabetes-induced photoreceptor damage (Fig. 3G). Gene enrichment analysis for the characteristic genes of the Müller cell 4 provided compelling evidence that supported the observations regarding the unique role of this cell type in the progression of DR. The BP enrichment analysis revealed a significant overrepresentation of genes associated with visual perception, response to stimulus, photoreceptor cell maintenance, and phototransduction. The overrepresentation of these processes in Müller cell 4 supported the hypothesis that these cells may be incorporating material from damaged rod photoreceptors through phagocytosis.

### Elucidating the role of Müller cells in the progression of DR

**Fig. 4:**
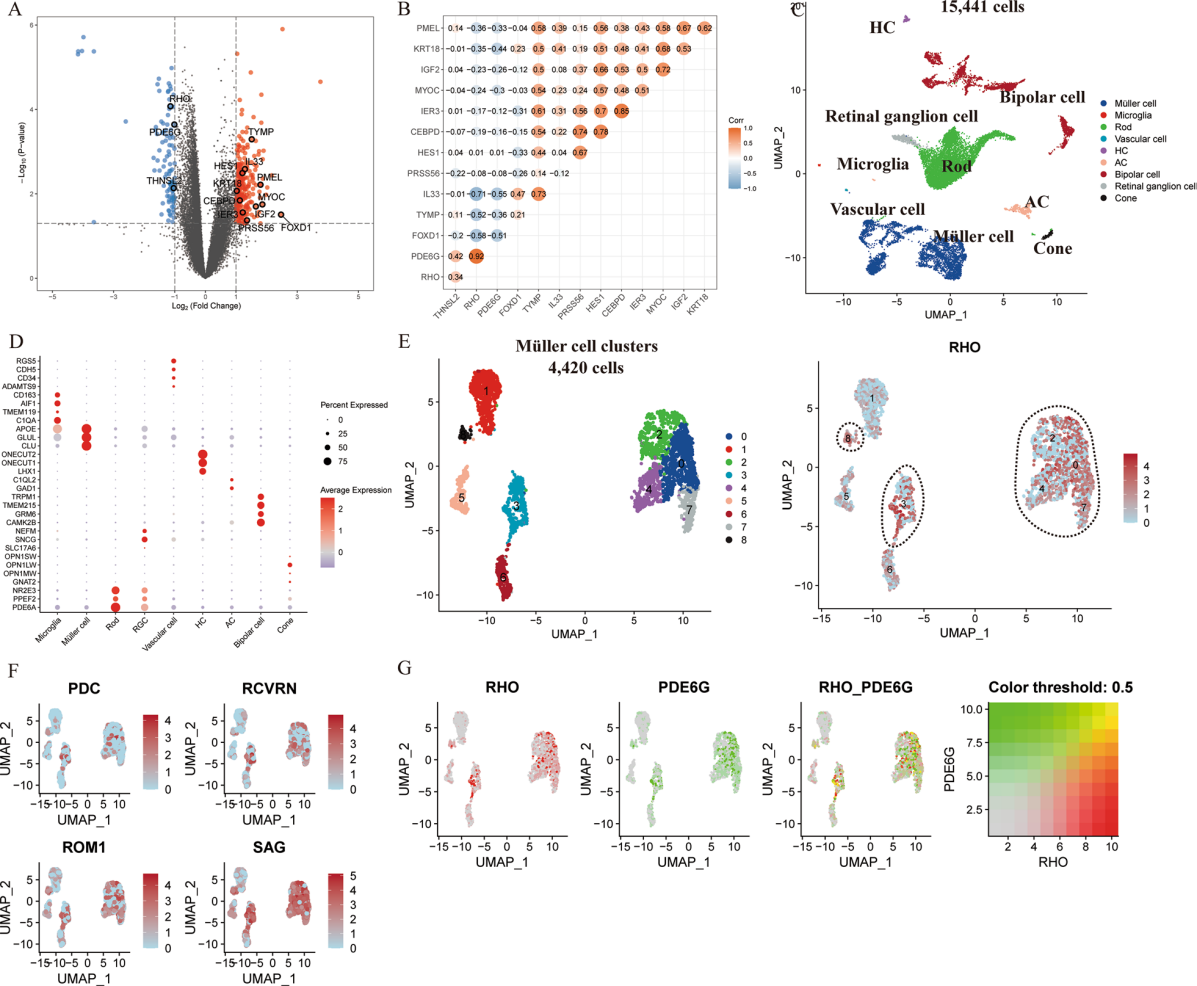
**A** The volcano plot of genes differentially expressed between PDR-DME and NPDR. Differentially expressed genes identified with monocle2 were labeled in the figure. **B** Gene co-expression regulatory network of intersecting genes. **C** The UMAP plot of human scRNA-seq data, colored for the 9 cell clusters. **D** The dotplot of the marker genes across individual cell clusters. **E** UMAP Visualization of Müller cell clusters and RHO expression. **F** UMAP plots showing the distribution of gene expression for the genes PDC, RCVRN, ROM1, and SAG across Müller cell clusters. **G** UMAP plots visualizing the expression patterns of RHO and PDE6G genes individually and their co-expression within Müller cell clusters

To elucidate the complex underpinnings of DR, meticulously dissecting the retinal cell atlas is imperative. Unfortunately, the integration of scRNA-seq with transcriptomic datasets for cross-species analyses remains an underexplored territory. Such integrative approaches are critical for a holistic understanding of the nuanced roles Müller cells play throughout the progression of DR. Addressing this gap, we undertook a differential gene expression analysis leveraging transcriptomic data from GSE160306. This rich dataset includes samples from individuals diagnosed with proliferative diabetic retinopathy with concurrent diabetic macular edema (PDR-DME) and those with NPDR, as depicted in Fig. 4A.

We correlated the genes displaying notable temporal changes, as identified through mouse pseudo-time analysis (Additional file 4: Table S2), with their corresponding human orthologs. This was followed by cross-referencing these genes with the outcomes of the differential gene expression analysis, as documented in Additional file 5: Table S3. Among the genes that emerged from this cross-referencing, we noted an upregulation in PDR-DME samples for TYMP, IL33, HES1, PMEL, KRT18, CEBPD, IER3, MYOC, IGF2, PRSS56, and FOXD1. Conversely, RHO, PDE6G, and THNSL2 exhibited downregulation in PDR-DME samples (Fig. 4A).

To unveil potential regulatory connections, we established a gene co-expression regulatory network involving these intersecting genes (Fig. 4B). The RHO gene was observed to have a significant positive correlation with the PDE6G gene, exhibiting a correlation coefficient of 0.92. This substantial correlation underscored the critical role of the RHO within the phototransduction pathway of the retina. As rhodopsin, encoded by the RHO gene, is integral to the visual process, its tight co-expression with PDE6G, which encodes a key component of the rod photoreceptor cGMP phosphodiesterase, highlighted the synchrony of these genes in visual function. The expression of RHO, typically restricted to rod photoreceptors, and its strong association with PDE6G in the dataset underpinned the specificity of these genes in rod photoreceptor operations. This interaction was further validated using the STRING database (Additional file 2: Fig. S2).

To validate our findings, we analyzed scRNA-seq data derived from the MR and PR of three patients each, encompassing a total of 15,441 cells, with a particular focus on identifying a spectrum of cell subtypes, including Müller cell populations (Fig. 4C–D). Figure 4E elucidates the diverse Müller cell clusters identified in the human samples. The UMAP on the right of Fig. 4E specifically illustrated the expression pattern of the RHO gene across these Müller cell clusters. Notably, the RHO gene expression was not homogeneously distributed across all Müller cell clusters. Instead, it showed a marked elevation in a subset of cells within certain clusters, highlighted by the dashed circles (Fig. 4E).

The upregulation of RHO in these Müller cell clusters may be indicative of an engagement with phagocytic activity, particularly concerning the components of damaged rod photoreceptors in the diabetic retina. This upregulation corroborated the hypothesis previously formulated based on single-cell data from SD rats (Fig. 4E).

Translating the characteristic genes identified in the Müller cell 4 from SD rats to their human orthologs, we assessed their expression levels within human Müller cell clusters. The UMAP plots depicted the expression patterns of these orthologous genes, including PDC, RCVRN, ROM1, and SAG, across the identified human Müller cell clusters. Remarkably, they exhibited localized upregulation in the same subsets of Müller cells where RHO expression was prominent. The observed gene expression trends provided additional evidence that certain Müller cell clusters, upon exposure to diabetes-induced retinal stress, may adaptively express genes that were not part of their typical repertoire. The consistent expression patterns between the rats and human data sets lent credence to the hypothesis that Müller cells are involved in preserving retinal integrity in the face of photoreceptor damage, highlighting the potential conservatism of this response mechanism across species (Fig. 4F).

Finally, we assessed the co-expression patterns of RHO and PDE6G within Müller cell clusters. The UMAP plots in Fig. 4G elucidated the co-expression patterns of RHO and PDE6G within Müller cell clusters in DR. Specifically, the concurrent expression of RHO, a gene exclusively expressed in rod photoreceptors, alongside PDE6G, a gene critical to the phototransduction process, within Müller cells suggested an atypical yet critical cellular phenomenon. Following the assessment of RHO and PDE6G co-expression within Müller cell clusters, we proceeded to validate the interaction between these two proteins with the STRING database. The visual output from the STRING analysis, as depicted in Additional file 2: Fig. S2, confirmed the interaction between RHO and PDE6G. The co-localization of these two genes within Müller cells posited a potential role for these glial cells in the phagocytic processing of rod photoreceptor components during DR. The role of Müller cells in mitigating the effects of diabetic retinopathy (DR) could be crucial, offering valuable insights into cellular resilience and the maintenance of retinal function under pathological conditions.

## Discussion

Our comprehensive analysis has uncovered valuable insights into the role of Müller cells in DR, shedding light on their potential contributions to the disease's pathology. The outcomes of our enrichment analysis distinctly reveal that the genes specific to Müller cell subtypes are significantly enriched in specific GO terms and KEGG pathways. This enrichment provides valuable insights into the functional characteristics and potential molecular mechanisms underlying Müller cells in the context of DR.

The enrichment of Müller cell-specific genes in GO terms such as membrane, plasma membrane, and protein binding suggests their involvement in essential cellular processes [17]. Müller cells are distinguished by their unique morphological and functional characteristics, including the formation of extensive radial networks within the retina. These processes interact with various neighboring cell types, such as neurons and vascular cells, indicating their active involvement in cell-to-cell communication and signaling [18]. The enrichment of genes related to membrane and plasma membrane functions highlights the importance of Müller cells in maintaining cellular homeostasis, regulating transport processes, and mediating interactions with surrounding cells [19–21].

In addition, the enrichment of Müller cell-specific genes in KEGG pathways, including metabolic pathways, pathways in cancer, and fluid shear stress and atherosclerosis, provides additional insights into the underlying molecular mechanisms. Müller cells are known for their metabolic activity and their essential role in energy metabolism within the retina [22]. The enrichment of genes in metabolic pathways suggests their involvement in processes related to energy production, nutrient uptake, and waste elimination. Furthermore, the association with cancer-related pathways suggests potential links between Müller cells and pathological processes, such as cell proliferation, survival, and migration, all of which are relevant in the context of retinal diseases [18, 23, 24]. Additionally, the enrichment of genes in the "Fluid shear stress and atherosclerosis" pathway suggests that Müller cells may respond to hemodynamic forces and participate in vascular remodeling and pathological angiogenesis, two distinctive features associated with DR [25, 26].

Overall, our findings from the enrichment analysis highlight the distinct transcriptional profiles and functional characteristics of Müller cell subtypes in DR. The specific enrichment of genes within particular GO terms and KEGG pathways emphasizes Müller cells’ involvement in fundamental cellular processes, metabolic regulation, interactions with neighboring cells, and their potential relevance to disease pathology. Further research is imperative to fully elucidate the precise roles and mechanisms governing the enrichment of Müller cell-specific genes within these functional categories.

Our study has provided valuable insights into the intercellular signaling and communication network of Müller cells, the primary glial cells in the retina known for their critical role in maintaining retinal homeostasis and responding to pathological changes [27–29]. Through our analysis of cell–cell interactions, we have unveiled distinct signaling pathways enriched in Müller cells, indicating their active involvement in intercellular communication within the retinal microenvironment.

Notably, pivotal factors such as PTN, MK, PSAP, and VEGF have emerged, with potential implications in DR. The PTN pathway, known for its diverse biological functions encompassing the regulation of cell proliferation, migration, and survival [30], could contribute to modulating Müller cell interactions with other cell types [31, 32]. Similarly, the MK pathway, associated with various cellular processes like cell–cell communication and tissue homeostasis [33], appears to be involved in intercellular communication among Müller cells, potentially influencing Müller cell function in the context of DR [34–36].

The VEGF pathway, a well-known regulator of angiogenesis, emerges as a critical mediator of communication between Müller cells and other cell types in the retina, such as BC, AC, HC [37]. Dysregulation of VEGF signaling in DR can lead to abnormal neovascularization and vascular leakage, thereby contributing to disease pathology [38, 39]. Understanding the significance of these signaling pathways in the context of Müller cell communication is essential for unraveling the molecular mechanisms underlying DR. Moreover, targeting these pathways presents promising therapeutic strategies to modulate Müller cell function and mitigate the effects of the disease.

Our findings from the scRNA analysis of Müller cells in DR presented novel insights into the temporal dynamics and cellular heterogeneity of these cells, which may have profound implications for our understanding of DR pathology and potential therapeutic strategies. The classification of Müller cells into six distinct subgroups and the observation of their dynamic changes over the course of DR (0 to 8 weeks) underscore the remarkable heterogeneity within this cell type.

The discovery of a bifurcated trajectory in pseudo-time analysis suggests that Müller cells may adopt two potential differentiation paths in response to the DR milieu. This finding challenges the conventional notion of Müller cells as a uniform population, emphasizing their adaptability and versatility under pathological conditions. Particularly intriguing is the unexpected high expression of Rho in Müller cell 4. Traditionally confined to rod photoreceptors, Rho's presence in Müller cell 4 suggests an unconventional response to DR, possibly indicative of cellular interaction or fusion events, such as phagocytosis of damaged rod cells. This hypothesis was supported by the dual expression of both rod-specific markers (Rho and Sag) and typical Müller cell markers (Glul and Apoe) within this subgroup.

The potential for Müller cells to engage in phagocytic activity, particularly under stress conditions, unveils new avenues for understanding the cellular mechanisms that contribute to retinal integrity and function in the disease state. Some research confirmed that Müller cells play a significant role in phagocytosis under various conditions. Müller cells, the primary glial cell type in the retina, are involved in phagocytosis of cell debris during the development of the visual system, and in pathological conditions, they can engulf apoptotic cell bodies and foreign substances. Additionally, the phagocytic activity of Müller cells has been observed to decrease as microglial cells become activated and migrate to the photoreceptor cell layer, suggesting communication between Müller cells, microglia, and photoreceptors.

Moreover, Müller cells have been shown to acquire proliferative activity in the damaged retina, indicating that engulfment of apoptotic photoreceptor debris might stimulate Müller glia to proliferate during the regenerative response [40–42]. Sanae Sakami confirmed that in a mouse model of retinitis pigmentosa, Müller cells are the primary cells responsible for phagocytosing dead rod photoreceptor cells, playing a key role in maintaining retinal homeostasis and preventing additional retinal damage during the progression of the disorder [43].

In the late stages of DR, the increased cellular homogeneity of Müller cells indicates a more synchronized and well-defined functional state [44]. By elucidating the temporal dynamics and cellular heterogeneity of Müller cells, we gain a better understanding of the mechanisms underlying DR [19]. This provides valuable perspectives for delving deeper into the interactions between Müller cells and other types of retinal cells, along with their participation in processes related to neuroinflammation, vascular dysfunction, and neural injury.

The integration of transcriptomic data analysis and scRNA-seq data analysis has provided a comprehensive understanding of gene expression dynamics in DR. This approach has afforded us valuable insights into the synchronized alterations occurring at the transcriptomic level within Müller cells. Among the upregulated genes, such as TYMP, IL33, HES1, and others, these genes have been associated with various BP, including inflammation, gliosis, extracellular matrix remodeling, and response to cellular stress [45–47]. The upregulation of these genes suggests their involvement in driving pathological changes within Müller cells, potentially contributing to the advancement and severity of DR.

Through the integrated analysis of scRNA-seq and transcriptomic data, we identified RHO and PDE6G as pivotal genes associated with DR. Prior research has indicated that these genes, classified as related to visual perception, hold a critical role in the development and progression of DR [48, 49]. Our study adopted a comprehensive approach, integrating human single-cell sequencing data, transcriptomic sequencing data, and rat single-cell sequencing data to validate the robustness of our findings.

Previous work by Kai Chen significantly contributed to characterizing the retinal cell atlas, utilizing a vast dataset that encompassed 276,402 cells from both humans and mice [14]. This research provided valuable insights into the early impacts of DR on retinal cells. Our study reveals that Müller cells, often considered mere supportive glial cells, actually assume a more active role in DR. They undergo significant transcriptional changes and demonstrated a marked upregulation of genes like RHO, traditionally associated with rod photoreceptors, suggesting a potential phagocytic activity towards damaged photoreceptor components. This alteration in gene expression patterns, particularly the co-expression of RHO and PDE6G, implies a novel and critical cellular phenomenon, indicating Müller cells' involvement in preserving retinal integrity under the stress of DR.

The clinical implications of our study go beyond identifying therapeutic targets; they offer profound insights into precision medicine strategies for DR. By unraveling the intricate molecular interactions within Müller cells and their responses to the diabetic milieu, our research opens new avenues for early diagnosis, disease monitoring, and prognostication. The differential expression patterns and complex interaction networks we uncovered serve as valuable biomarkers for identifying DR at its onset, tracking disease progression, and predicting patient outcomes.

Moreover, the signaling pathways we identified pave the way for developing novel pharmacological interventions aimed at modulating Müller cell functions to either prevent or arrest the progression of DR. Our cross-species analysis highlights the conservation of certain molecular mechanisms between rodents and humans, enhancing the translational potential of our findings. This aspect is crucial for validating the relevance of our models and the applicability of our molecular insights to human diabetic retinopathy.

By integrating multi-omics data, our study unraveled the intricate complexities surrounding Müller cells in DR. Through a cross-species analysis, we explored the diverse transcriptional profiles of Müller cells across different stages of the disease, shedding light on their heterogeneity. Utilizing protein–protein interaction analysis, we identified novel molecular interactions that could play pivotal roles in the pathogenesis of DR. These findings challenge conventional perceptions, highlighting Müller cells as essential regulators crucial for maintaining retinal homeostasis and influencing disease progression. Our study not only enriches the molecular understanding of DR, but also presents promising avenues for targeted therapeutic interventions. By elucidating the molecular intricacies within Müller cells, our work paves the way for developing precise therapies aimed at preserving vision in individuals affected by diabetes.

## Conclusion

In conclusion, our study provides a comprehensive exploration of retinal cellular responses in diabetic retinopathy, emphasizing the crucial involvement of Müller cells in disease progression. The revelation of Müller cells' participation in phagocytic activities, particularly their interaction with damaged rod photoreceptor components, represents a significant breakthrough in our comprehension of DR at the molecular level. Moreover, the identification of key hub genes and their associated pathways offers promising avenues for potential therapeutic interventions. These findings underscore the importance of recognizing the dynamic and multifaceted roles of retinal cells, particularly Müller cells, in devising effective treatments for DR. Our research significantly contributes to the broader understanding of DR pathophysiology and sets the stage for future investigations aimed at mitigating the impact of this vision-threatening condition.

### Supplementary Information

**Additional file 1: Fig. S1.** The dot plot illustrates the significant signaling pathways and ligand–receptor pairs, where the dot color indicates the probabilities of communication and the dot size depicts the corresponding p-values.

**Additional file 2: Fig. S2.** The interaction between RHO and PDE6G identified with STRING database.

**Additional file 3: Table S1.** Enrichment Analysis Results.

**Additional file 4: Table S2.** Differentially expressed genes along the pseudo-time.

**Additional file 5: Table S3.** Human and Rat Gene Name Correspondence.

## Data Availability

The data used in this study were retrieved from three GEO datasets available in the GEO database: GSE209872, GSE137537 and GSE160306.
