# Supplementary figures and images for: Deciphering Müller cell heterogeneity signatures in diabetic retinopathy across species: an integrative single-cell analysis
Source: Eur J Med Res. 2024 May 3;29:265. doi: 10.1186/s40001-024-01847-y (PMC11067085; doi:10.1186/s40001-024-01847-y)

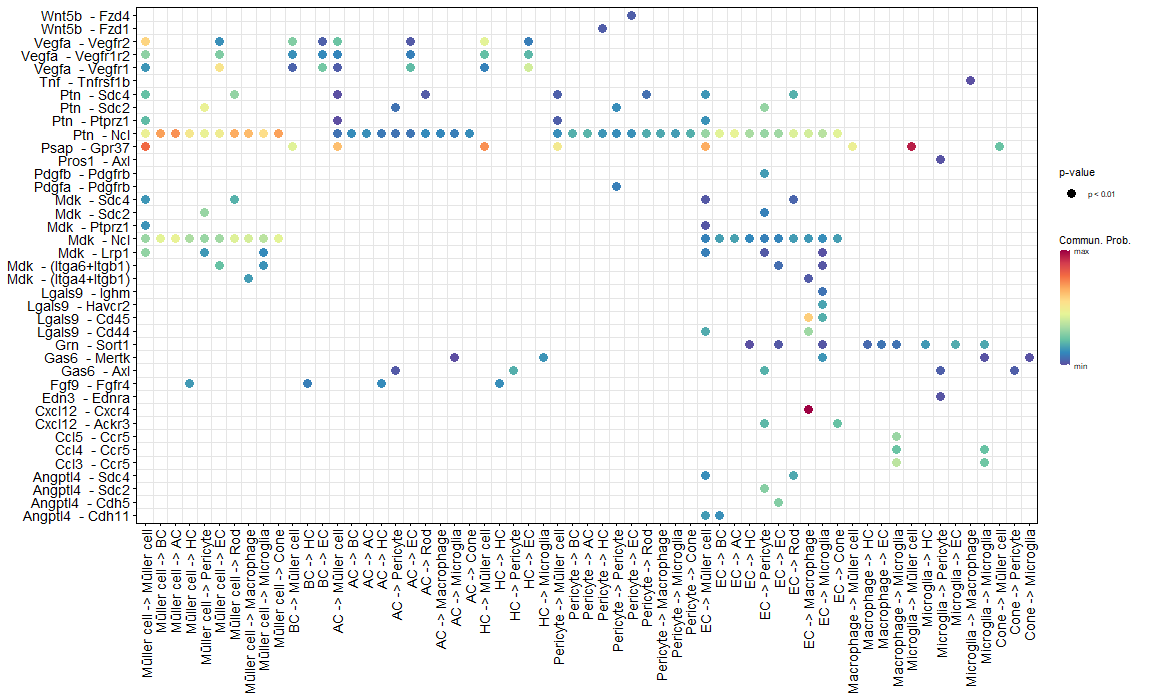

Supplement: Supplementary file 1 — Additional file 1: Fig. S1. The dot plot illustrates the significant signaling pathways and ligand–receptor pairs, where the dot color indicates the probabilities of communication and the dot size depicts the corresponding p-values. [file 40001_2024_1847_MOESM1_ESM.tif]

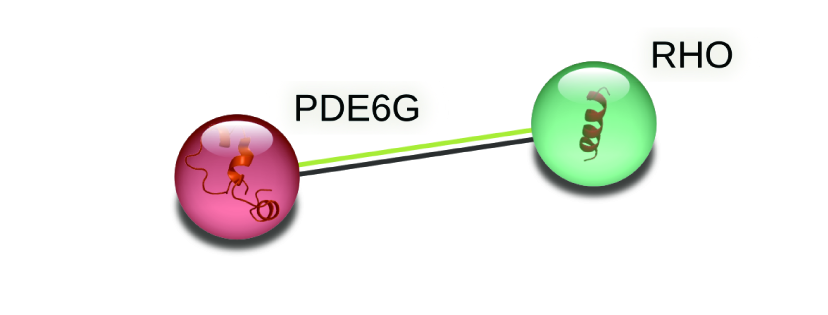

Supplement: Supplementary file 2 — Additional file 2: Fig. S2. The interaction between RHO and PDE6G identified with STRING database. [file 40001_2024_1847_MOESM2_ESM.tif]
